# The cognitive structure of psychiatry prior to COVID-19: a bibliometric analysis

**DOI:** 10.1192/j.eurpsy.2026.10943

**Published:** 2026-07-31

**Authors:** P. Vicente, A. L. Terra, J. Johnston

**Affiliations:** 1 University of Coimbra, CEIS20 — Centre for Interdisciplinary Studies, Faculty of Arts and Humanities, Coimbra, Portugal; 2 University of Iceland, Reykjavík, Iceland

## Abstract

**Introduction:**

Several studies have utilised bibliometrics to analyse scholarly literature and uncover the cognitive structure of the domain of psychiatry over the course of the COVID-19 pandemic and in its aftermath; however, the pre-pandemic period remains overlooked. Amid the COVID-19 crisis, it was claimed that “the COVID-19 pandemic is changing the face of psychiatry permanently”, “the shift in psychiatric research will undoubtedly affect scientific publishing in psychiatry” (Türközer and Öngür Mol Psychiatry; 25 2214–2219).

**Objectives:**

Therefore, our aim was to present a conceptual map of psychiatric scholarly output published before COVID-19.

**Methods:**

For the bibliometric analysis, 28 journals in the top quartile of the Psychiatry category in the 2019 edition of the Journal Citation Reports were analysed, including *European Psychiatry.* Bibliographical records of journal articles for the period 2014–2018, prior to the emergence of COVID-19, were exported from the Web of Science citation database. Author keywords were used as the units of analysis, on which a term co-occurrence analysis was conducted. The cognitive structure of the domain of psychiatry was drawn from the terminological and conceptual structure extracted from author keywords and visualised using the VOSviewer software.

**Results:**

A corpus of 12,294 journal articles, encompassing 17,341 distinct author keywords, was analysed. Figure 1 shows the term co-occurrence network among author keywords in the analysed psychiatry journal articles. The network is observed to centre around the concept “depressive disorder,” which shares the strongest link in the whole network with the concept “anxiety disorders,” suggesting that depressive disorder and anxiety disorders are concepts commonly addressed together in the scholarly literature of psychiatry prior to COVID-19. There is also a robust link between “depressive disorder” and “bipolar disorder,” and, naturally, with “antidepressant,” the class of pharmaceutical drugs usually administered to treat depressive disorders. The concept “bipolar disorder” also shares a strong link with “schizophrenia,” and “schizophrenia” with its primary class of pharmaceutical drugs, “antipsychotic”.

**Image 1:**
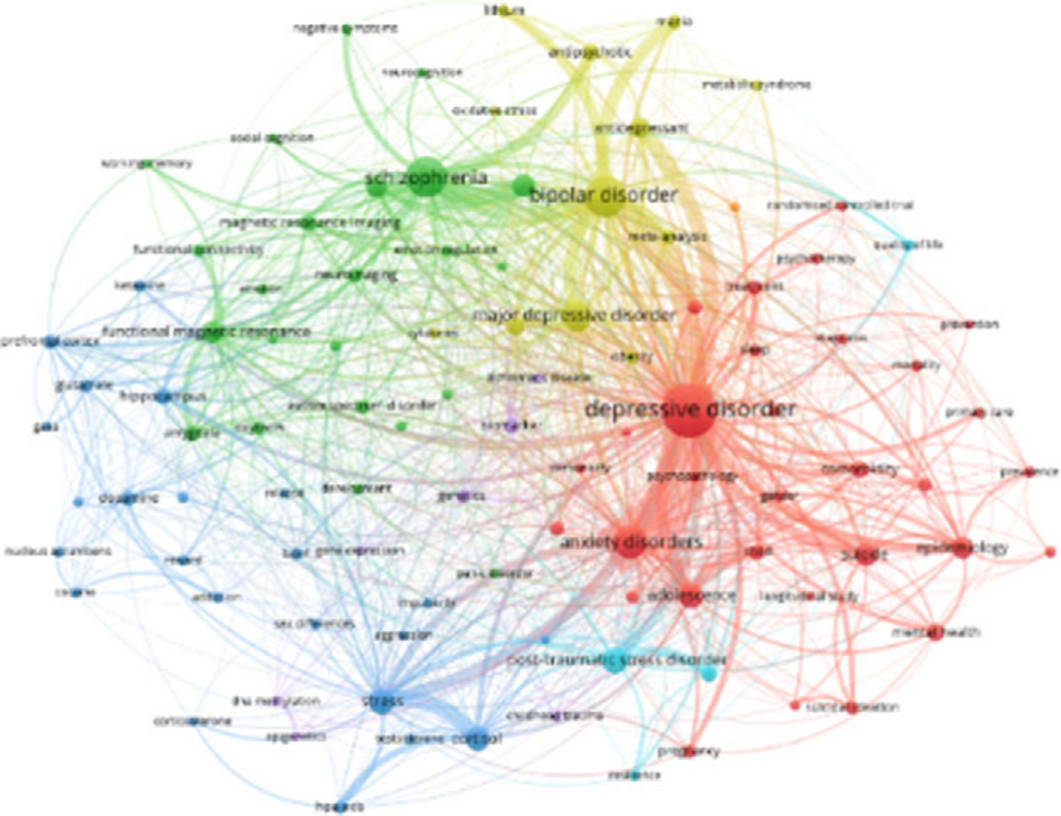
Image 1: Long description.

**Conclusions:**

Beyond the core mental disorders that constitute the cognitive structure of psychiatry, the presence of other concepts, such as brain structures and biomolecular compounds, suggests that, during the period analysed, psychiatric scholarship was primarily framed within a biomedical model. Furthermore, studies of the psychiatric scholarly literature during the COVID-19 pandemic and its aftermath have consistently identified depression and anxiety as focal concepts; however, this pattern already characterised the pre-pandemic period. Nonetheless, bipolar disorder and schizophrenia were pivotal concepts before the pandemic, yet their prominence fell sharply during the pandemic, signalling a significant shift in focus.

**Disclosure of Interest:**

None Declared

## Long descriptions

### Image 1: Long description

A network diagram consisting of nodes and connecting lines of varying thickness. The nodes are grouped into four main color-coded clusters.

* Red Cluster (Right): Centered on ‘depressive disorder’ and ‘anxiety disorders’. Peripheral nodes include ‘suicide’, ‘epidemiology’, ‘comorbidity’, ‘psychotherapy’, and ‘adolescence’.

* Green Cluster (Top-Left): Centered on ‘schizophrenia’ and ‘functional magnetic resonance imaging’. Connected terms include ‘working memory’, ‘prefrontal cortex’, ‘neuroimaging’, and ‘social cognition’.

* Yellow Cluster (Top-Center): Centered on ‘bipolar disorder’ and ‘major depressive disorder’. It bridges the red and green clusters with terms like ‘antidepressant’, ‘antipsychotic’, ‘lithium’, and ‘meta-analysis’.

* Blue Cluster (Bottom-Left/Center): Centered on ‘stress’ and ‘cortisol’. It includes biological and physiological terms such as ‘dopamine’, ‘hippocampus’, ‘post-traumatic stress disorder’, ‘genetics’, and ‘h p a axis’.

The density of lines is highest between ‘depressive disorder’, ‘anxiety disorders’, and ‘bipolar disorder’, indicating strong thematic overlap in the research data.

Navigate back to Image 1:.

